# A Multilayer Emitter Close to Ideal Solar Reflectance for Efficient Daytime Radiative Cooling

**DOI:** 10.3390/polym11071203

**Published:** 2019-07-18

**Authors:** Yeqing Zhu, Dong Wang, Cheng Fang, Ping He, Yong-Hong Ye

**Affiliations:** 1 Department of Physics, Nanjing Normal University, Nanjing 210023, China; 2 Department of Information Engineering, Nanjing Normal University Taizhou College, Taizhou 225300, China

**Keywords:** thin film, solar reflectance, emissivity, daytime radiative cooling, thermal-radiation, cooling performance

## Abstract

A passive radiative cooling method has a significant influence on thermal management applications because it can cool without any energy input. This work both experimentally and theoretically demonstrates a multilayer thin film structure with high solar reflectance, which can be applied to passive daytime radiative cooling. The combination of physical vapor deposition and spin-coating prepared the samples, which were also characterized experimentally by spectrometers. On-site measured results show that the emitter can effectively achieve daytime radiative cooling, and the cooling performance can be further improved with the increase of the ambient air temperature. When the emitter is exposed to direct solar radiation (AM1.5) of about 880 W/m^2^ on a rooftop under dry air conditions, it can achieve an average temperature reduction of about 12.6 °C from the ambient air temperature with nonradiative heat transfer (11 a.m.–1 p.m.). Theoretical simulations reveal that the emitter can still have a certain cooling performance in the presence of significant nonradiative heat exchange and nonideal atmospheric conditions. The influence of ambient air temperature on the cooling performance of the emitter is also theoretically analyzed.

## 1. Introduction

Passive radiative cooling systems have been extensively studied in the past. Since ancient times, it has been well known that black emitters facing a clear night sky can achieve sub-ambient temperatures [1]. Efficient night-time radiative coolers with promising infrared emissivity have been broadly investigated in both organic and inorganic materials [2,3,4]. However, due to the influence of solar absorbance in daytime, it is still a challenge for daytime radiative cooling. Recently, daytime radiative cooling under direct solar radiation has been reported, where a specially designed emitter radiates energy through the atmospheric transparency window (8–13 μm), and reflects most of the incident sunlight. As a successful example, Zhai et al. [5] proposed a daytime cooling structure where a visibly transparent polymer hybrid metamaterial was embedded with randomly distributed SiO_2_ microspheres. When backed with a silver (Ag) substrate, the metamaterial has the properties of scalable selective radiation. It is found that the type, the size, the volume fraction, the order of microspheres, the polymer materials, and reflective substrates will directly affect the cooling performance of the structure. Other theoretical and experimental works in passive radiative cooling structures based on microspheres embedded in a matrix have also been reported [6,7,8,9]. Additionally, some periodic nanostructures can also be used to implement passive radiative cooling, including photonic crystals [10], metamaterials [11,12,13], metallic photonic crystals [14,15], and so on. Apart from the works discussed above, some review papers [16,17,18,19,20,21] also introduce radiative cooling systems.

In the meantime, recent works have shown that multilayer thin film structures can also be used to achieve daytime radiative cooling under direct sunlight. The designed structures emit around 8 to 13 μm and reflect visible light [22,23,24,25,26,27]. Raman et al. [22] presented a radiative cooler consisting of seven alternating layers of HfO_2_ and SiO_2_ on top of a 200 nm thick Ag substrate as the back reflector, which results in 3% of sunlight absorption. They experimentally demonstrated that the radiative cooler was able to cool about 5 °C below ambient air temperature (*T*_a_) under direct sunlight (AM1.5) of about 860 W/m^2^ on a rooftop. Kou et al. [23] demonstrated a thin and simple near-black IR emitter of a fused silica wafer coated with polydimethylsiloxane (PDMS) on top and Ag on the back, which could achieve a temperature reduction of about 8.2 °C from *T*_a_ under direct sunlight on a rooftop. They also suggested that the cooling performance could be improved by reducing sunlight absorption in the ultraviolet region. However, this idea has not been experimentally demonstrated. This paper aims to experimentally demonstrate an improved multilayer thin film structure, which can remarkably reduce the absorption of solar radiation in the ultraviolet region.

## 2. Materials and Methods

### Sample Design and Preparation

**Figure 1:**
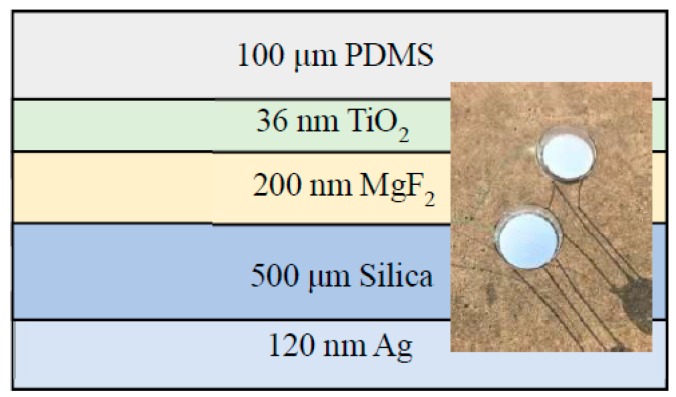
(Color online) Schematic of multilayer thin film structure for efficient daytime radiative cooling. (Inset) Image of the radiative emitters on the test rooftop in Nanjing, China.

By using the needle optimization technique, the number of layers and layer thicknesses of the emitter are determined and optimized [28]. The target metrics chosen for optimization are ideal solar albedo around 0.3–4 μm and ideal emissivity around 4–25 μm. The calculation of the model is dependent on admittances of each layer and optical thickness of the structure. Thickness, the refractive index, and angles of incidence are considered as inputs, and spectroscopic performance of the structure can be obtained. The simulations are performed in the Essential Macleod in order to better investigate the spectroscopic performance of the emitter. The refractive indices and extinction coefficients of SiO_2_ and MgF_2_ used in the simulations are all derived from the Essential Macleod software. The optical properties of Ag (0.3–25 μm), TiO_2_ (0.3–25 μm), and PDMS (2.5–25 μm) are all from COMSOL, with other values for PDMS coming from Reference [29]. Two samples are prepared for comparison. Emitter 1 is the proposed structure to improve solar reflectance (*R*_sol_). The design of the improved radiative emitter 1 is shown in Figure 1. It consists of five layers including a coating of a 200-nm thick MgF_2_, a 36-nm thick TiO_2_, and a 100-μm thick PDMS film on top of a 4 inch fused 500-μm thick silica wafer, respectively, and a 120-nm thick Ag film on the back. The MgF_2_ and Ag layers were deposited by thermal evaporation, while the TiO_2_ layer was fabricated by electron beam evaporation. During the deposition, the thickness of each layer was monitored by a quartz crystal monitor. Lastly, a 100-μm thick PDMS film was spin-coated on top of the TiO_2_ layer for 60 s, which was followed by degassing for 10 min and curing for 1 h at 80 °C [23]. Emitter 2 is close to Kou’s structure [23], with a coating of the 100-μm thick PDMS layer on top of a 4 in. fused 500 μm thick silica wafer, and 120-nm thick Ag on the back layer. Testing is done on the flat roof of a six-story building in Nanjing, China (Figure 1, inset). SiO_2_ has a strong absorption peak at the atmospheric transparency window near 10 μm due to the existence of its phonon-polariton resonance [22], which makes it attractive for usage in passive radiative cooling devices. The insertions of MgF_2_ and TiO_2_ layers are equivalent to vary the refractive-index profile of the multilayer thin film structure. Interference effects associated with these two materials will optimize the reflectivity of the sunlight. PDMS is a silicone elastomer, which is remarkably transmissive between 0.4–1.8 μm and is easy to deposit [23,30]. In our case, PDMS is designed to counteract the impedance mismatch between the silica and air around 8–13 μm [23]. Due to the high reflectivity of Ag from the visibility to the IR regions, *R*_sol_ is maximal in these wavelengths. In general, the integration of all the materials creates a macroscopically planar structure that is able to achieve high *R*_sol_ from 0.3–4 μm and strong thermal emissivity for IR regions longer than 4.5 μm.

## 3. Results and Discussion

### 3.1. Spectroscopic Performance

**Figure 2:**
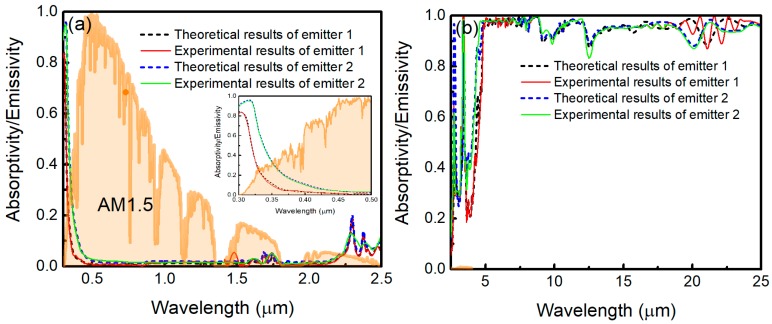
(Color online) (**a**) Measured emissivity/absorptivity spectra of emitter 1 (red solid curve) and emitter 2 (green solid curve) at 12° angle of incidence by using an unpolarized light source from ultraviolet to near-IR wavelengths, with the normalized AM1.5 solar spectrum plotted for Reference [31]. Under the same conditions, theoretical results for emitter 1 (black dashed curve) and emitter 2 (blue dashed curve) are plotted for comparison. (Inset) Zoom-in of the measured and theoretical results for emitter 1 and emitter 2 in the ultraviolet region. (**b**) Measured emissivity/absorptivity spectra of emitter 1 (red solid curve) and emitter 2 (green solid curve) at 30° angle of spectra incidence by using an unpolarized light source over mid IR wavelengths. Theoretical results for emitter 1 (black dashed curve) and emitter 2 (blue dashed curve) are plotted for comparison.

**Figure 3:**
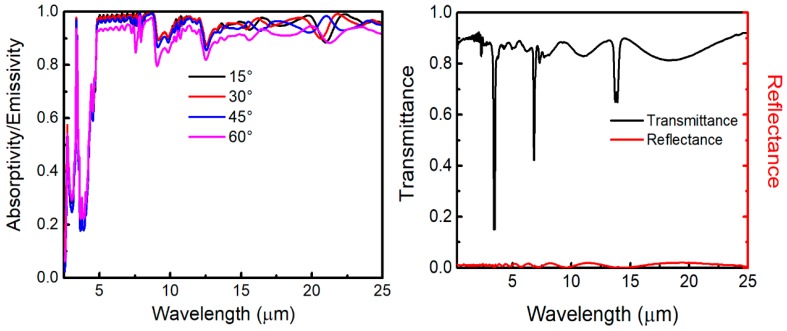
(Color online) (**a**) Measured angular emissivity/absorptivity of emitter 1 from 2.5 to 25 μm. (**b**) Spectral transmittance and reflectance of the polyethylene film.

The absorptivity/emissivity spectra of the emitters measured by using an UV-Vis-NIR Spectrophotometer (Cary 5000, Varian, Salt Lake City, UT, USA, at 12° angle of incidence from ultraviolet to near-IR wavelengths), and Fourier transform infrared spectrometer (FTIR, Nicolet Nexus 670, GMI, Bunker Lake Blvd. Ramsey, NJ, USA, at 30° angle of incidence over mid-IR wavelengths) are shown in Figure 2a,b, respectively. In the measurements, an unpolarized light source is used. As can be seen in Figure 2, the theoretical results are in good agreement with our experimental results. Comparing with emitter 2, the sunlight absorption of emitter 1 is decreased, especially in the ultraviolet region, which is shown in Figure 2a (inset). The sunlight absorption power density for emitter decreases from 21 to 10.3 W/m^2^, which shows the improved *R*_sol_ by adding the layers of MgF_2_ and TiO_2_. In the meantime, the absorption is minimal from visible to near IR wavelengths. Figure 2b shows that the emissivity of the two emitters approach unity from 4.5 to 25 μm due to the absorption of SiO_2_ and PDMS [23]. Figure 3a depicts the measured emissivity of emitter 1 at varying incidence angles from 15° to 60° in near and mid IR regions. Clearly, the thermal emissivity is insensitive to the incidence angles. It is a useful feature to maximize the radiated power. In the testing, an 18-μm thick low-density polyethylene film is chosen as an infrared-transparent wind shield. The measured spectral properties are shown in Figure 3b. The polyethylene film is not perfectly transparent, and its transmittance is considered in the next theoretical calculations.

### 3.2. On-Site Measurements

**Figure 4:**
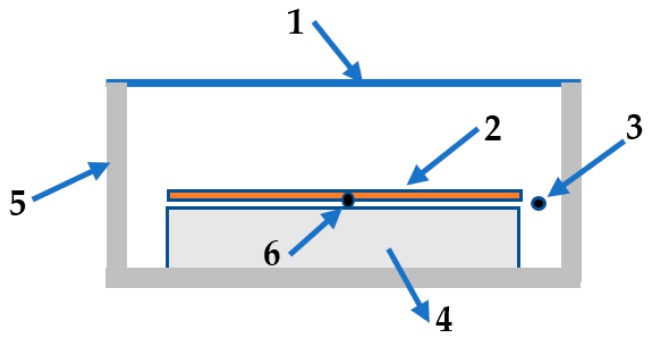
(Color online) Schematic drawing of the test instrument. (**1**). 18-μm thick low-density polyethylene film. (**2**). Multilayer emitter. (**3**). K-type thermocouple for measuring the ambient air temperature. (**4**). 5-mm thick aerogel blanket. (**5**). Petri dish. (**6**). K-type thermocouple for measuring the emitter temperature.

**Figure 5:**
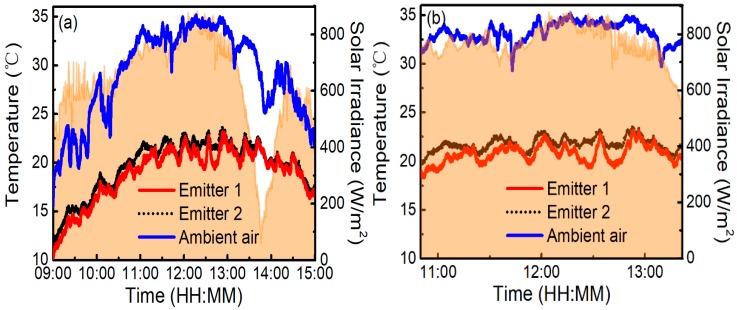
(Color online) (**a**) On-site measured temperatures of emitter 1 (red solid curve), emitter 2 (black dotted curve), and ambient air (blue solid curve) between 09:00 and 15:00 on the flat roof in Nanjing, in mid-October 2018. The orange shaded regions represent solar irradiance. (**b**) Zoom-in of on-site measured temperatures of emitter 1 (red solid curve), emitter 2 (black dotted curve), and ambient air (blue solid curve) from about 11:00 to 13:00.

To demonstrate the cooling performance of the radiative emitters, on-site measurements have been conducted on the flat roof of a six-story building in Nanjing, in mid-October 2018, by exposing the two emitters to a clear sky during daytime and comparing the steady-state temperatures of the structures with *T*_a_. Figure 4 shows the schematic cross-section of the test instrument. Each emitter is placed flat on a 5-mm thick low thermal conductivity aerogel blanket attached to the inside of the Petri dish. The diameter of the Petri dish is 120 mm. The Petri dish is supported by three rods to ensure a certain height from the roof, and the height of the support rods is 23.5 cm. An 18 μm thick low-density polyethylene film is placed 2 cm above the emitter, which acts as an infrared-transparent wind shield and represents the experimental demonstration of the efficient daytime radiative cooling. The steady-state temperatures of the radiative emitter and *T*_a_ are detected by K-type thermocouples with ±0.5 °C accuracy, as labeled in Figure 4 [32]. The thermocouple is anchored with conductive cement at the center of the emitter’s backside, which is connected to a data logger (ATEST Thermometer DT-847UD, GODEE, Guangzhou City, China). The data are recorded every second. During the test period, the relative humidity is 20% to 70% and local wind speed is 0–1.5 m/s. A peak total solar irradiance of 880 W/m^2^ is plotted in Figure 5. All the data are derived from the Meteorology Bureau of Nanjing City. Figure 5a reveals that, even though there is a significant solar irradiance on the samples, the temperatures of both emitters can drop below *T*_a_. The cooling performance of emitter 1 is better than that of emitter 2. Figure 5b demonstrates that emitter 1 maintains an average temperature reduction of about 12.6 °C from *T*_a_ with solar radiation and nonradiative heat transfer between about 11:00 and 13:00 (local time). The daytime temperature differential of emitter 1 is about 1.0 °C larger than that of emitter 2.

**Figure 6:**
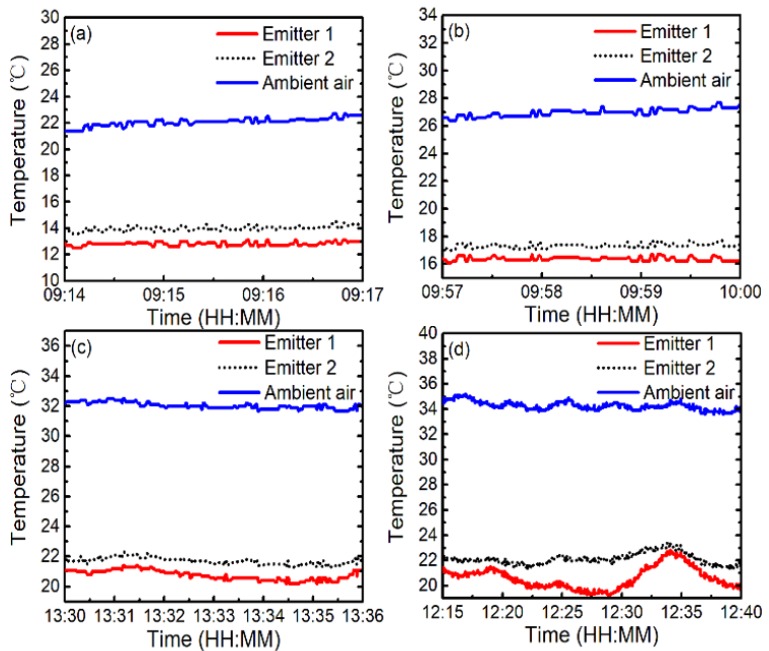
(Color online) Zoom-in of on-site measured results of emitter 1 (red solid curve), emitter 2 (black dotted curve), and ambient air (blue solid curve) in different temperatures with solar radiation and nonradiative heat transfer. The ambient air temperature is about: (**a**) 22 °C, (**b**) 27 °C, (**c**) 32 °C, and (**d**) 35 °C.

To concretely analyze the effect of the ambient air temperature on cooling performance, we zoom in some results in Figure 5a. Figure 6 illustrates zoom-in of on-site measured temperatures of emitter 1 (red solid curve) and emitter 2 (black dotted curve), with solar radiation and nonradiative heat transfer at *T*_a_ (blue solid curve) of about 22, 27, 32, and 35 °C, respectively. As can be seen in Figure 6a, emitter 1 can maintain an average temperature reduction of about 9.3 °C from *T*_a_ (about 22 °C) in the daytime operation, while the temperature reduction of emitter 2 is about 8.2 °C. When *T*_a_ is about 27, 32, and 35 °C, the temperature differential of emitter 1 is about 10.7, 11.5, and 12.6 °C, while that of emitter 2 is about 9.7, 10.5, and 11.5 °C, as shown in Figure 6b,d respectively. Therefore, the cooling performances of the emitters grow as *T*_a_ increases.

### 3.3. Solar Irradiation and Atmospheric Transmittance

**Figure 7:**
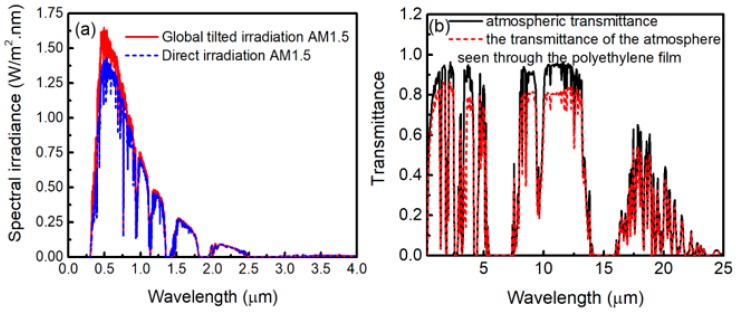
(Color online) (**a**) The global tilted and direct irradiation of the AM1.5 spectrum [31]. (**b**) The spectral transmittance of the atmosphere from MODTRAN 5 [34].

To explain the measured results of the radiative cooling emitters, theoretical calculations are performed. It is necessary to consider the solar irradiation and atmospheric transmittance for daytime radiative cooling. The global solar irradiation flux is about 1000 W/m^2^, while, for a clear sky, the diffuse reflection component is about 50–100 W/m^2^ [33]. The standard average solar irradiation is usually represented by the solar spectrum of AM1.5, which is shown in Figure 7a [31]. The irradiance of AM1.5 global tilt spectrum is about 964 W/m^2^ [12]. Figure 7b shows the atmospheric transmittance in the zenith direction t(λ) (black solid curve) and the atmospheric transmittance in the zenith direction seen through the polyethylene film $t^{\prime }\left(\lambda \right)$ (red dashed curve). The black solid curve is obtained from MODTRAN 5 [34], which is defined as a clear sky transmittance in mid-latitude winter. In the modeling, some atmospheric parameters are as follows. Aerosol of urban visual range is 23 km, seasonal modifications to aerosol are fall-winter, no clouds or rain, and the surface range for the boundary layer is 20 km. The atmospheric emissivity seen through the polyethylene film is $\varepsilon^{\prime }\left(\lambda ,\theta \right)=1-t^{\prime }{\left(\lambda \right)}^{1/cos\theta }.$ In Reference [35], $\varepsilon^{\prime }\left(\lambda ,\theta \right)$ is determined by the equation $\varepsilon^{\prime }\left(\lambda ,\theta \right)=\varepsilon \left(\lambda ,\theta \right)+\varepsilon_{PE}-\varepsilon_{PE}\varepsilon \left(\lambda ,\theta \right)$, where $\varepsilon \left(\lambda ,\theta \right)=1-t{\left(\lambda \right)}^{1/cos\theta }$ is the atmospheric emissivity and $\varepsilon_{PE}$ is the emissivity of the polyethylene film. Moreover, $\varepsilon_{PE}$ is insensitive to the angles when θ< 45° [35]. In general, a body’s reflectivity (*r*), transmittance (*t*), and absorptivity (α) are related as r+t+α=1. By using Kirchhoff’s radiation law, for anything at thermal equilibrium, absorptivity equals to emissivity. Thus, $\varepsilon_{PE}=1-r_{PE}-t_{PE},$ where $r_{PE}$ and $t_{PE}$ are the reflectivity and transmittance of the polyethylene film, respectively. $\varepsilon_{PE}$ can be taken from the measured results in Figure 3b.

### 3.4. Theoretical Cooling Performance and Discussion

**Figure 8:**
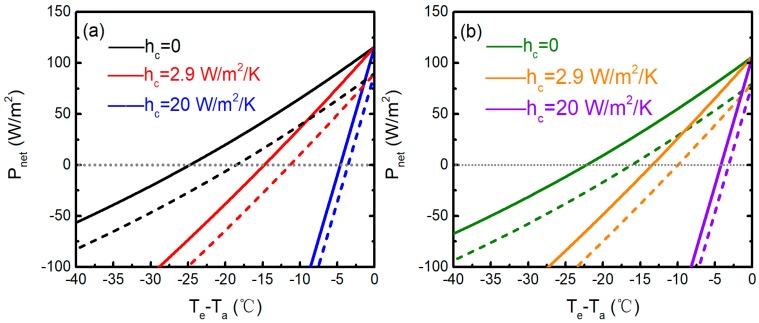
(Color online) (**a**) Calculated net cooling power of emitter 1 as a function of the temperature difference for non-radiative heat transfer coefficient *h*_c_ of 0 (black curves), 2.9 (red curves), and 20 W/m^2^/K (blue curves), in high (solid curves) and low (dashed curves) atmospheric transmittance during daytime operation. (**b**) Calculated net cooling power of emitter 2 as a function of temperature difference for non-radiative heat transfer coefficient *h*_c_ of 0 (olive curves), 2.9 (orange curves), and 20 W/m^2^/K (violet curves), in high (solid curves) and low (dashed curves) atmospheric transmittance during the daytime operation. The *T*_a_ is assumed to be 300 K.

The net cooling powers of the emitters per unit area *P*_net_(*T*_e_-*T*_a_) given $T_{a}=300\;K$ in daytime for various values of non-radiative heat transfer coefficient *h*_c_ in both high *(*t*)* and low *(*$t^{\prime }$*)* atmospheric transmittance are plotted in Figure 8. *T*_e_ represents the surface temperature of the emitter, and the data of the emissivity are the measured ones in Figure 2. The theoretical results are obtained by usual weighted integrations from the spectra of *R*_sol_, *t*(*λ*), and sky radiation absorptance *A*_sky_ [12]. By considering the experimental location (Nanjing city) in October, the incident angle is assumed to be 30°. Figure 8 shows the calculated net cooling power as a function of the temperature difference of emitters for different *h*_c_ and atmospheric transmittance. The negative values for *T*_e_-*T*_a_ demonstrates that the structures have the abilities to cool down below the ambient air temperature in daytime operation. Figure 8 depicts that emitter 1 (blue curves) and emitter 2 (violet curves) can both achieve daytime radiative cooling performance even at $h_{c}=20\;W/m^{2}/K$. In this case, as shown in Figure 8a, emitter 1 can achieve an average temperature reduction of 4.6 and 3.6 °C from *T*_a_ in high (blue solid curve) and low (blue dashed curve) atmospheric transmittance during daytime operation, respectively, and yield net cooling powers of 116.0 and 89.9 W/m^2^, respectively. Meanwhile, Figure 8b shows emitter 2 can cool down 4.2 and 3.1 °C below *T*_a_ in high (violet solid curve) and low (violet dashed curve) atmospheric transmittance during daytime operation, respectively, and yield the net cooling powers of 106.1 and 79.9 W/m^2^, respectively. Therefore, the emitters can achieve a significant daytime cooling performance even with moderate wind-induced convection and conductive heat exchange. It is known that nonradiative heat exchange and nonideal atmospheric conditions are two significant factors in passive radiative cooling performance. Figure 6b illustrates that emitter 1 can maintain an average temperature reduction of about 10.7 °C from *T*_a_ (about 27 °C) during the daytime operation, while the daytime temperature differential of emitter 2 is about 9.7 °C. The experimental results match with the theoretical ones effectively in high atmospheric transmittance, as shown in Figure 8. When $h_{c}=2.9\;W/m^{2}/K$, emitter 1 (red solid curve) and emitter 2 (orange solid curve) can theoretically cool down 11.3 and 10 °C from *T*_a_ in high atmospheric transmittance during the daytime operation, respectively. The value of *h*_c_ = 2.9 W/m^2^/K is the result of a fit to the experiment. Thus, the polyethylene film encapsulating the emitters can basically eliminate wind-induced convection and non-radiative heat conduction. Due to the high solar reflectance of the emitters, the requirements for the cover material is reduced when comparing with previously proposed structures. It is noteworthy that the peak solar irradiance of 880 W/m^2^ is the raw irradiance. Due to the high solar reflectance of the emitters from ultraviolet to near-IR wavelengths, the corrections for the optical properties of the polyethylene film are negligible.

**Figure 9:**
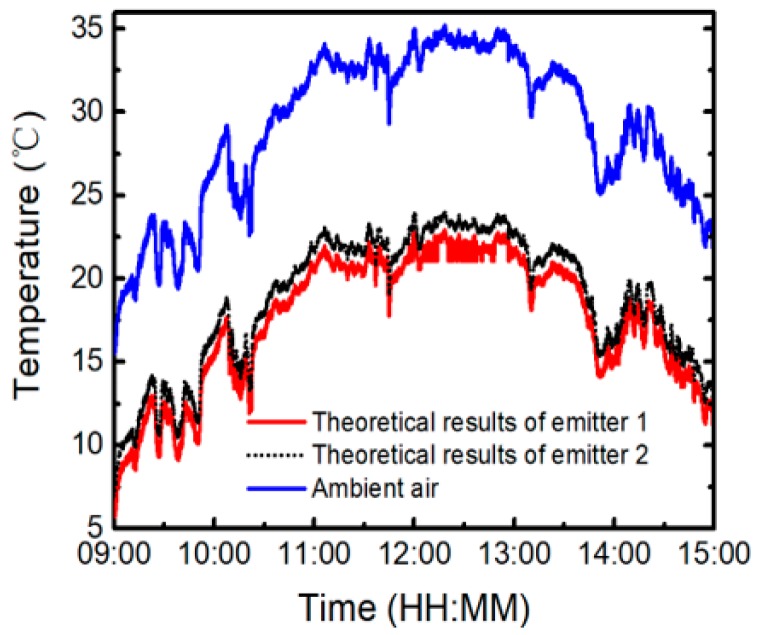
(Color online) The modeled temperatures of emitter 1 (red solid curve), emitter 2 (black dotted curve), and ambient air (blue solid curve, the same data as in Figure 5a) between 09:00 and 15:00.

**Figure 10:**
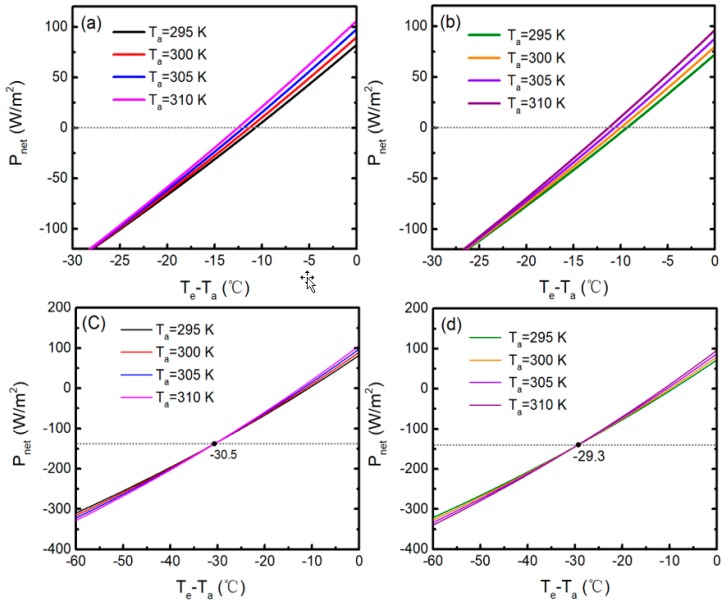
(Color online) Calculated net cooling power as a function of the temperature difference of emitters when *T*_a_ is 295, 300, 305, and 310 K in daytime operation: (**a**) emitter 1, results from −30 to 0 °C, (**b**) emitter 2, results from −30 to 0 °C, (**c**)emitter 1, results from −60 to 0 °C, (**d**) emitter 2, results from −60 to 0 °C. The nonradiative heat exchange is assumed to be 2.9 W/m^2^/K.

As can be seen from Equation (1) in Reference [14], *P*_net_ is a function of *T*_e_ and *T*_a_. In previous studies, *T*_a_ is usually assumed as a specific value while analyzing the radiative cooling performance of selective emitters, and the radiative cooling abilities in various ambient air temperatures have never been quantitatively discussed before [5,12,14,22,23]. Even in the summer, the diurnal temperature difference can be as high as 20 °C, depending on its location. Therefore, it is important to study the effects of *T*_a_ on cooling performance. Figure 9 shows the modeled temperatures of the two emitters at varying *T*_a_ (the experimental data in Figure 5a), with solar radiation and $h_{c}=2.9\;W/m^{2}/K$. The emissivity and transmittance properties of the polyethylene film are considered in the theoretical model. It can be seen that the modeled results are in good agreement with the measured ones in Figure 5a. The discrepancies near 9:00–9:50, 14:30–15:00, 10:10–10:20, and 13:40–14:10 are primarily due to the high relative humidity and the effect of the cloud, respectively. To quantitatively explain the measured results in Figure 6, the theoretical calculations are depicted in Figure 10. As can be seen from Figure 10, changes in *T*_a_ have an indispensable effect on the radiative cooling performance of the emitters. The cooling power can be further improved with the increase of *T*_a_. The increase of *T*_a_ will increase both the thermal radiation of the emitter and the thermal radiation absorbed from the atmosphere. However, the increase of the former is greater than that of the latter, and the solar absorption is unaffected by *T*_a_. Thus, the radiative cooling performance eventually improves. For emitter 1, when $T_{e}-T_{a}>-30.5\;{}^{\circ}C$, the higher *T*_a_ is, the better radiative cooling performance is, which matches well with the experimental results in Figure 6. Otherwise, the results are contrary. The transition temperature differential of emitter 2 is about −29.3 °C. The detailed calculated cooling performance of the two emitters at different *T*_a_ is summarized in Table 1. It is proven that, under the same conditions, emitter 1 is more effective due to the optimization of *R*_sol_.

## 4. Conclusions

In conclusion, a macroscopically planar multilayer thin film emitter for efficient daytime radiative cooling is both theoretically and experimentally demonstrated. Comparing with the previous work, the added MgF_2_ and TiO_2_ thin film layers effectively increases the solar reflectance in the ultraviolet region, which improves the cooling performance. The mature technique of manufacturing the emitter discussed in this case sets the stage for large-scale fabrication. There are many applications of passive radiative emitter in various temperature-sensitive optoelectronic devices, such as thermophotovoltaics, rectennas, photovoltaics and infrared detectors, which will stimulate the continuous interests of photonic film structures, thermal nano-photonics, and metamaterials.

## Figures and Tables

**Table 1:** Calculated cooling performance on $T_{a}$, *h*_c_ = 2.9 W/m^2^/K.

$T_{a}\left(K\right)$	$\Delta T_{1}\left({}^{\circ}C\right)$	$P_{net1}(W/m^{2})$	$\Delta T_{2}\left({}^{\circ}C\right)$	$P_{net2}(W/m^{2})$
280	−8.9	62.0	−7.4	51.7
285	−9.5	68.4	−8.0	58.2
290	−10.1	75.2	−8.7	65.1
295	−10.7	82.3	−9.3	72.3
300	−11.3	89.9	−10.0	79.9
305	−11.9	97.8	−10.6	87.9
310	−12.7	106.1	−11.3	96.3
